# Dynamic modeling of female neutering interventions for free-roaming dog population management in an urban setting of southeastern Iran

**DOI:** 10.1038/s41598-022-08697-w

**Published:** 2022-03-21

**Authors:** Saeedeh Shamsaddini, Milad Ahmadi Gohari, Hossein Kamyabi, Saeid Nasibi, Ali Derakhshani, Mohammad Ali Mohammadi, Seyed Mohammad Mousavi, Mohammad Reza Baneshi, Elly Hiby, Majid Fasihi Harandi

**Affiliations:** 1 Research Center for Hydatid Disease in Iran, Kerman University of Medical Sciences, 76169114115 Kerman, Iran; 2 Modeling in Health Research Center, Institute for Futures Studies in Health, Kerman University of Medical Sciences, Kerman, Iran; 3 Department of Medical Parasitology, School of Medicine, Kerman University of Medical Sciences, 7616914115 Kerman, Iran; 4 Faculty of Medicine, Centre for Longitudinal and Life Course Research, School of Public Health, The University of Queensland, Herston, QLD 4006 Australia; 5 The Alliance for Contraception in Cats and Dogs (ACC&D), Cambridge, UK

**Keywords:** Ecological modelling, Urban ecology

## Abstract

Understanding dynamics of free-roaming dog (FRD) population is critical for planning and implementation of dog population management programs. FRD population size estimation as well as dynamic modeling of dog population under different female dog neutering interventions were investigated in order to determine the most appropriate animal birth control approach. We performed population size estimate of dogs using sight-resight surveys by photography in a randomly selected 25 blocks of the city and all the suburbs of greater Kerman area. Main demographic features were characterized and the dog density distribution was mapped. A dynamic model was developed to predict free-roaming dog population variations after 5 and 10 years. Different scenarios based on 10, 30, 50, 60 and 70% female dog sterilization were considered to predict the effects of animal birth control measures. Free roaming dog population was estimated at 6781 dogs (65.3% males) in Kerman and suburbs with several major population hotspots. Analysis of the dog locations within the city showed that the largest proportion of the dogs were observed in the vacant lots (46.2%). Modeling predictions indicated that, in the absence of management, the free-roaming dog population could increase from a baseline of 6781 to 13,665 dogs (2.02 fold increase) in 5 years and to 19,376 dogs in 10 years (2.86 fold increase). Using a population dynamics model, we simulated five neutering coverages to explore the impact of female neutering on free-roaming dog population size. The 5-year projections of the model have shown that 50% annual female dog sterilization significantly reduced free-roaming dog population by 0.44 comparing to the baseline population. Findings of the present study improve our knowledge on the nature and extent of dog population dynamics in Iran. Effective population control and selection of the most appropriate neutering interventions require a comprehensive knowledge of the characteristics and dynamics of FRD population.

## Introduction

Dogs were the first animals domesticated about 15,000 years ago. Based on genetic studies and fossil data, the dog’s genetic separation from the wolf occurred less than 100,000 years ago. Interactions between humans and dogs may influence the health of individuals, families and communities^1^. The favorable impact of human–dog interactions encompasses a wide variety of socioeconomic and cultural roles that dogs perform in societies as companion animals as well as conducting certain special duties^2,3^. However because of different causes, many countries witness an increasing population of Free-Roaming Dogs (FRDs). The nature of these factors are different depending on the socioeconomic features of the societies. In high-income countries lack of responsible ownership, abandonment and escape or loss of pet dogs are among the main causes of increasing FRD population, while in low/middle income countries, FRD populations are on the rise due to poor population management schemes and a cultural barrier and perceptions towards the adoption of FRDs^4,5^. FRDs are potentially capable of transmitting several zoonotic infections to humans including cystic echinococcosis^6^, rabies^7^, visceral leishmaniasis^8^ and soil transmitted helminthes^9^. In addition traffic accidents and environmental health issues are associated with free-roaming dog population^10^. Dog population management (DPM) is a major social and health challenge in many urban and rural areas of developing countries.

Main approaches in FRD population management include dog culling, fertility control, and long-term sheltering. The ethical concerns as well as negative public reactions caused many countries to stop using culling. When comparing different interventions, fertility control may most greatly reduce free-roaming dog population and improve animal welfare^11,12^. The efficacy and impact assessment of different FRD populations management plans should be considered in terms of reducing dog population density, improving public health and animal welfare, and performance of DPM facilities^13,14^.

Dog population management should be based on appropriate community-based strategies and be focused on promoting community health, animal welfare, and reducing the impact of free-roaming dogs on the predation of livestock and native wildlife. Community participation is central to any DPM intervention^15^ and is essential for sustainable solution of human–dog conflicts. Instances of the successful community engagement can be found in the literature. For example in Dehradun and Vadodara, India, successful DPM programs with a fertility control approach implemented with a particular focus on community engagement^16,17^. In Dehradun, appropriate interaction between the communities and DPM program resulted in 16% reduction in overall complaints about dogs and 80% decrease in request for relocation of dogs^17^. Practical planning and effective population control and selection of the most appropriate populational control strategies require a comprehensive knowledge of the demographic characteristics and dynamics of FRD populations^10^. Estimation of dog population size and spatial distribution of the dogs in urban areas are essential to evaluate intervention measures.

Different methods of population size estimation have been used in different parts of the world. Census counting, distance-based methods as well as capture-recapture surveys have been used to determine the size of free-roaming dog populations. More recently several studies have used sight-resight method to estimate the size of dog populations^18^. Among numerous studies on FRD population size estimate around the world, capture-recapture methods have been used in the Philippines, Spain, Nepal, USA, Japan, India and Nigeria^19–23^. In Sorsogon province of the Philippines, dog population estimate was performed by capture-recapture methods using plastic, light-colored water-resistant collars and found a density of 468 dogs per km^2^^24^. As a prerequisite for the implementation of dog population management programs, Tiwari et al. used sight-resight method by photography to estimate demographic composition of FRDs in north India^25^. The study showed that sight-resight is a quick and relatively low-cost method for generating demographic data for FRDs. Photography-based sight-resight methods are advantageous compared to methods that require the capture and handling of dogs. Photographic capture is simpler, safer, requires lower costs and reduces handling-related risks to dog health and welfare. In addition, researchers are less likely to be bitten by free-roaming dogs and exposed to the zoonotic pathogens. However the method is landscape-dependent and high quality photography is not always possible. Also interpreting the images is sometimes difficult particularly when photographing several dogs in a pack^26,27^. Identification and reidentification of dogs with less distinctive colors or markings can be challenging, and there is a potential for mis-identification, which can reduce accuracy of results.

Animal population growth is determined by parameters related to the reproduction, mortality and migration^28^. Our understanding of the dynamics of FRD populations including spatial distribution and population size in the Middle East and North Africa is limited. Several models have been developed to evaluate the impact of dog population management interventions, particularly fertility control programs. In Brazil dog population size was estimated and population dynamics was modeled using demographic parameters^29^.

In Jodhpur, India dog population size was shown significantly declined in three of five areas following an animal birth control (ABC) program^30^. However several important parameters have to be considered in an optimized ABC program. Using a multi-compartment time series model, Hogasen et al. investigated the parameters affecting the dynamics of the dog population in Italy and examined the impact of different management choices on FRD population size over a period of 10 years^31^. Sterilization rate as well as age- and sex-related interventions are among the important parameters, however few modeling studies have investigated the impact of these parameters on DPM interventions. A study on simulating DPM in Brazil showed the need to increase the current sterilization rates^29^. Female dogs in reproductive age are central to any FRD population control program. Findings of a study performed in Mexico suggested that sterilization of young female dogs can enhance reductions in owned dog population size in a 20-year horizon, compared to the current sterilization strategy focused on dogs, regardless of age and sex^32^. Nonetheless little is known on the impact of sterilizing unowned female dogs population. Targeting female dogs in ABC programs is crucial, as shown by Yoak et al. that the fertility control method targeting areas of the city of Jaipur with the highest percentage of intact female FRDs outperforms other fertility control and lethal programs in reducing dog population size. They demonstrated that lethal methods skew dog population towards younger dogs, and increase the frequency of conflict with humans^33^. Therefore different demographic and socioeconomic aspects of dog population are essential for a successful DPM program. Belsare and Vanak examined the time, effort, financial resources, and conditions needed to successfully control FRDs in an urban setting in India. They developed a model tool to simulate processes and challenges of dog population management and to understand the expected measures needed to control the FRD populations in the region^34^.

In Iran, information on free roaming dog population is very poor and no studies have been conducted on dog population estimate. Only rough estimates have been made using the ratio of dog:human population according to the World Association for the Protection of Animals and the World Health Organization in 1990^35^. Keeping dogs as companion animals is not popular in the country due to the social/cultural factors. However in recent years increasing number of families are using dogs as pet animals particularly in larger metropolitan cities. The owned dogs mostly include sheep and guard dogs and due to the lack of responsible ownership and very low rate of dog adoption, many puppies abandoned in the city and suburbs, leading to the increasing human–dog tensions due to high number of unowned free roaming dogs. Nonetheless the authors field observations indicate very few owned roaming dogs are also present in the city mostly belonged to the homeless people in the streets and public parks. Frequent dog bites have caused negative perceptions and ill feeling towards dogs in the society. The number of animal bites (> 90% dog bites) in Iran have been estimated at 230,019 cases between 1993 and 2013 with an overall incidence of 13.2 per 1000 population^36^. Unfortunately no comprehensive dog population management has ever been implemented in Iran and systematic Trap (Capture)-Neuter-Vaccinate-Release (TNVR/CNVR) programs have not been established in the country, however some initiatives are being made by non-governmental organizations (NGOs) and municipalities in large cities. In several cities dog shelters have been provided by NGOs and a very small proportion of FRDs are neutered monthly, nonetheless the TNVR programs are not implemented properly and the neutered dogs are not released in their original locations. Therefore the implementation of perfect DPM system in Iran is in its infancy and the limited interventions have been generally made without evidence of their effectiveness and/or without an understanding of the dog population dynamics. The purpose of this study was to estimate the population size of free roaming dogs and to study the dynamics of the dog population using modeling methods in order to determine the most appropriate DPM strategies based on female dog neutering interventions under different levels of coverage.

## Results

**Figure 1:**
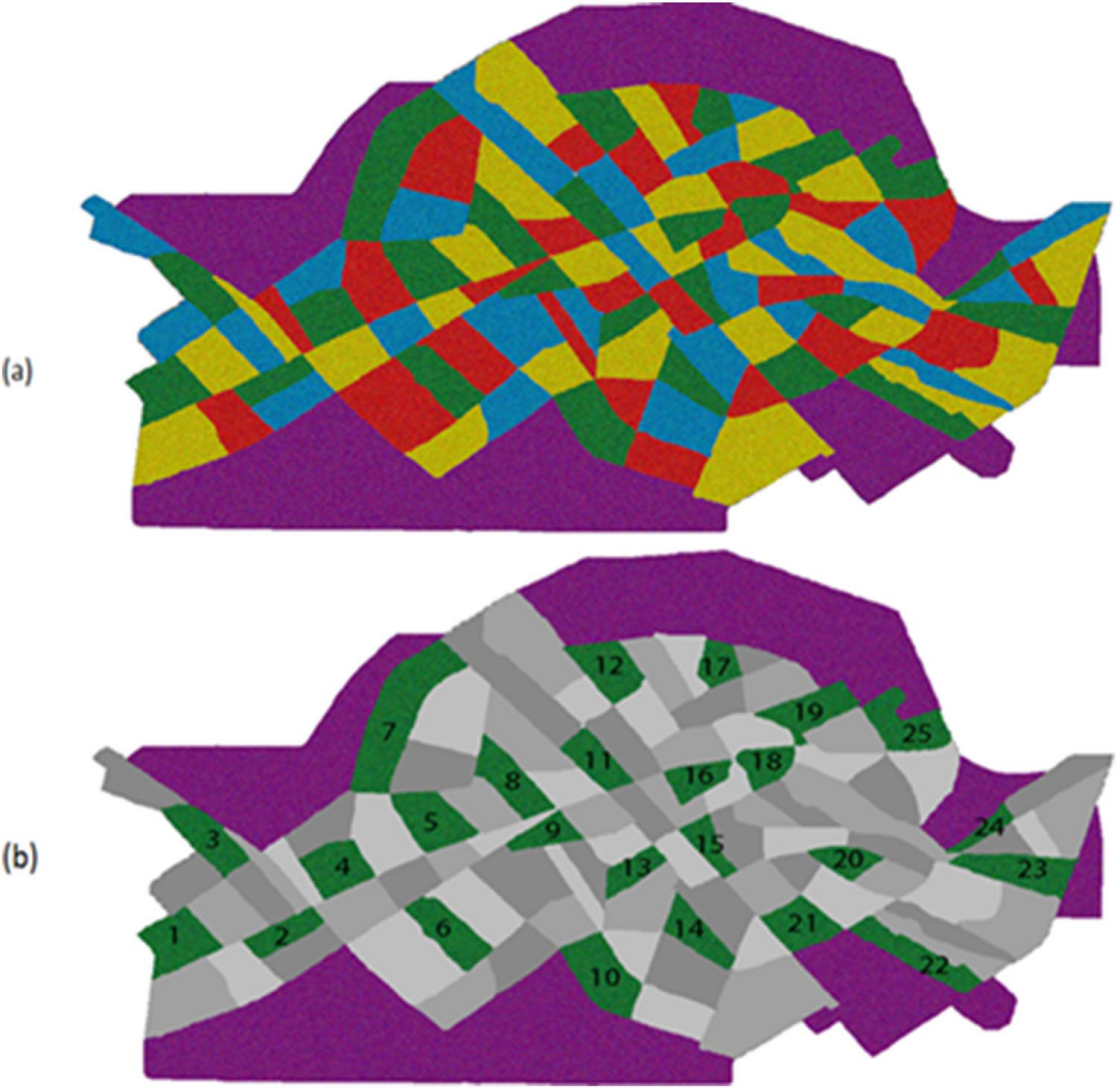
(**a**) Schematic representation of the suburbs and city of Kerman, Iran, showing the city divided into 100 blocks. (**b**) All selected green and purple blocks indicate the areas selected for street survey using sight-resight method for free-roaming dog population estimate.

Twenty-five blocks in the city and 15 suburbs were explored for FRD population (Fig. 1). Free roaming dog population was estimated at 6781 dogs that means 1.2 dogs per 100 people, 5.8 dogs per km street survey and a population density of 30.8 dogs/km^2^. The human:dog proportion was estimated at 80.7:1. In 25 selected city blocks, 283 and 272 dogs were recorded in capture and recapture surveys, respectively. These figures were 635 and 505 dogs for suburban areas. In the city and suburbs 83 and 103 dogs were re-observed, respectively. In total 1509 dogs including 986 (65.3%) males, 303 (21.1%) females and 220 (13.6%) unknown were recorded during sight-resight surveys across the city and suburbs. Table 1 shows the comparison of the demographic characteristics of free-roaming dog populations between the city and suburbs of Kerman.

**Table 1:** Multiple logistic regression analysis of the characteristics of free-roaming dog population between the city and suburbs of Kerman, presented as odd ratios (OR) and 95% confidence intervals (CI).

Demographics***	Suburb (%)	City (%)	Univariate analysis		Multivariate analysis*		Multivariate analysis**	
			Odds ratio (95% CI)	P-value	Odds ratio (95% CI)	P-value	Odds ratio (95% CI)	P-value
**Sex**								
Male	698 (78.7)	279 (71.0)	1		1		1	
Female	189 (21.3)	114 (29.0)	1.5 (1.1, 2)	< 0.01	1.6 (1.2, 2.1)	< 0.01	1.59 (1.20, 2.11)	< 0.01
**Age**								
Pup/young	121 (13.6)	39 (9.9)	1		1		–	
Adult	766 (86.4)	354 (90.1)	1.4 (1, 2.1)	0.06	1.3 (0.9, 1.9)	0.21	–	
**Body condition**								
Thin/emaciated	164 (18.5)	6 (1.5)	1		1		1	
Ideal/overweight	723 (81.5)	387 (98.5)	14.6 (6.4, 33.4)	< 0.01	14.9 (6.5, 34)	< 0.01	15.03 (6.58, 34.31)	< 0.01
**Social organization**								
Pair	178 (20.1)	65 (16.5)	1		1			
Single	156 (17.6)	65 (16.5)	1.1 (0.8, 1.7)	0.52	1.2 (0.8, 1.8)	0.45	–	
Pack	553 (62.3)	263 (66.9)	1.3 (1, 1.8)	0.1	1.2 (0.9, 1.7)	0.20	–	

*Logistic regression with Enter/ Forward method.

**Logistic regression with Backward method.

***Respectively, sex, age, and body condition of 220, 10 and 14 FRDs were missing.

The results of multiple logistic regression analysis are shown in Table 1. Female dogs are significantly more likely to be seen in the city than the suburbs (OR = 1.6, 95% CI 1.2–2.1, P < 0.01). FRDs were more likely to have a normal/overweight than a thin/emaciated body condition in the city compared to the suburbs (OR = 14.9, 95% CI 6.5–34, P < 0.01). No significant difference were observed in the odds of sighting young dogs between the city and suburbs. The social organization of the dogs did not show any difference between the two areas (Table 1). In total 37.0% of female dogs were pregnant or lactating, however the proportions were not significantly different between the city (36.8%) and suburbs (37.2%).

**Figure 2:**
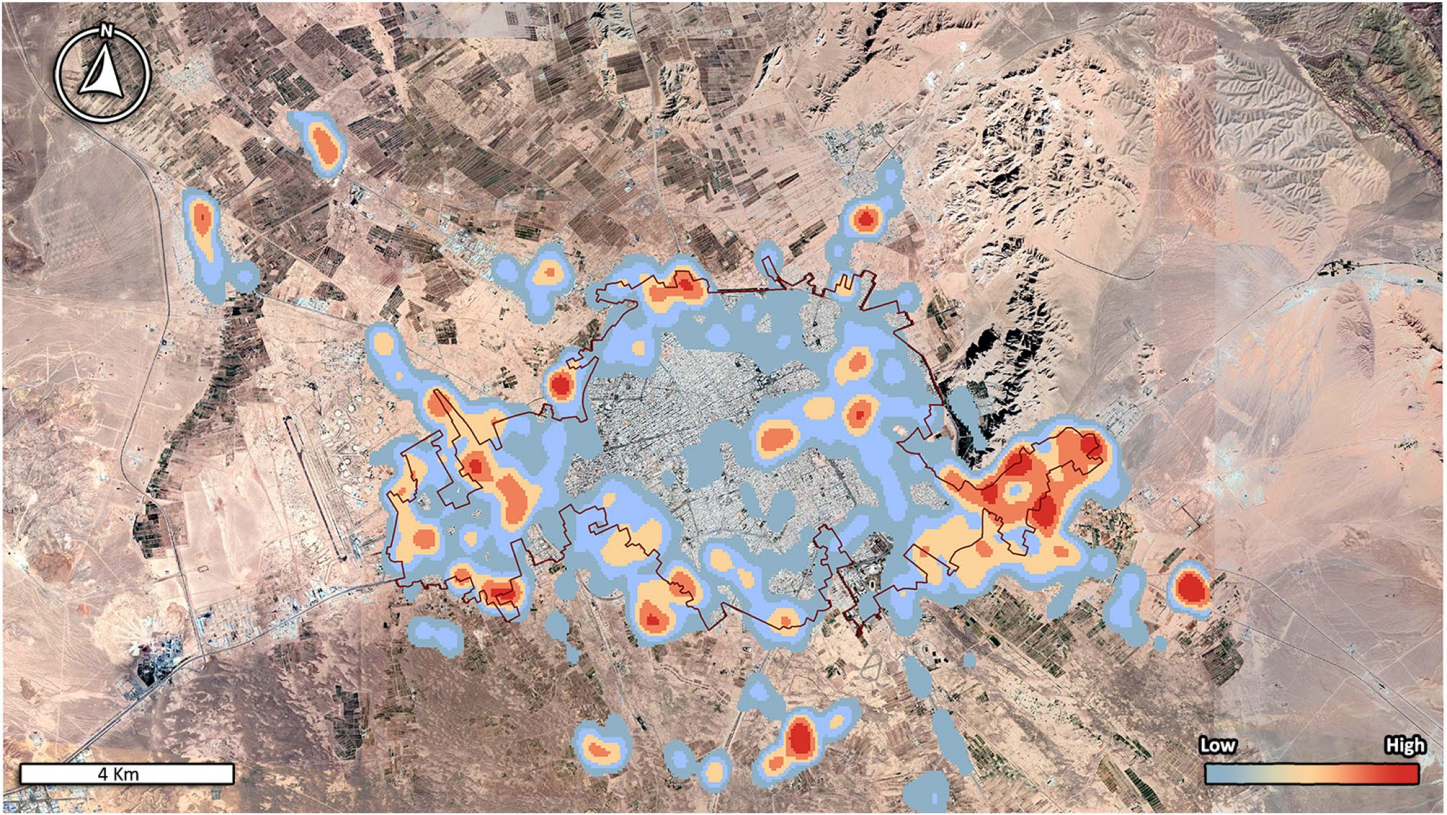
Estimated free-roaming dog population density in Kerman city and suburbs, Iran. This map was generated by ArcGIS 10.2 software with the Kernel technique (https://www.esri.com/en-us/arcgis/products/arcgis-pro/overview) using Google Earth Pro 7.3.4.8248 (earth.google.com).

Free roaming dogs were shown to be concentrated in certain city locations, indicating a significant distribution difference among various locations (χ^2^ = 316.03, P < 0.0001). Analysis of the dog locations within the city showed that the largest proportion of the dogs were observed in the vacant lots (46.2%) followed by the streets/sidewalks (24.4%) and alleys (24.4%). Only 4.2% and 0.8% of FRDs were found in public parks and next to the restaurants and garbage bins, respectively. Figure 2 shows the density of FRD population in a distribution map, demonstrating several major hotspots of FRD population inside the city and suburbs.

**Figure 3:**
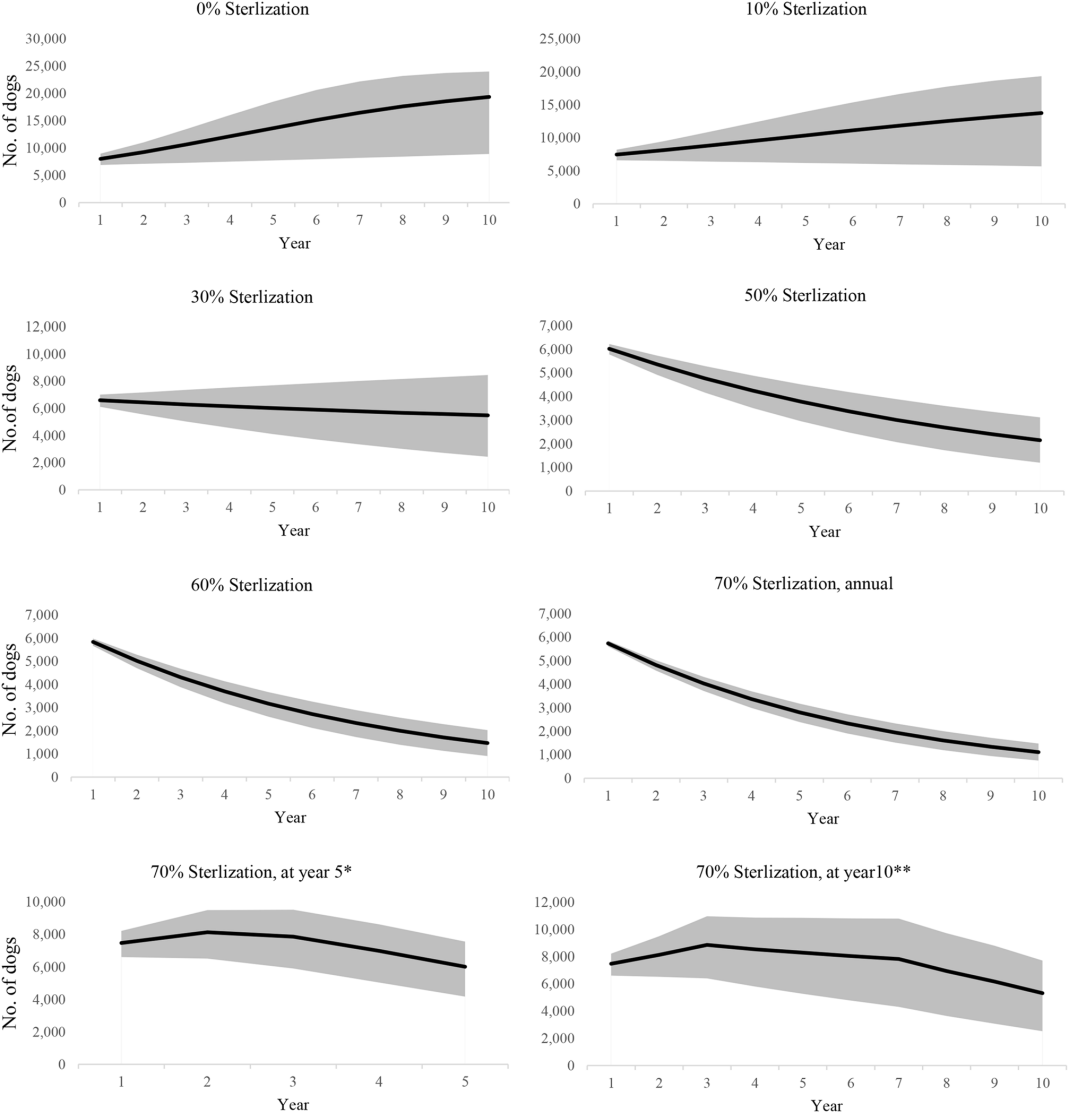
Dog population model predictions according to five different scenarios of animal birth control in a 5- and 10-year intervention program (*Consistent spaying of dogs over 5 years so as to cover 70% of the females in the final year. **Consistent spaying of dogs over 10 years so as to cover 70% of the females in the final year).

Figure 3 shows the dynamics of dog population in the city and suburbs. Results of the dynamic modeling under no intervention scenario indicate a dog population increase of 2.02 and 2.86 times in a 5- and 10-year period, respectively. It means FRD population increases from the baseline of 6781, to 13,665 and 19,376 dogs within 5 and 10 years after the present time. For dog population management, six scenarios were developed in the modeling to predict changes in FRD population in the region. Table 2 demonstrated the six prediction models in FRD population comparing five scenarios of dog population control intervention. The results indicated that with greater female neutering coverage, the decrease in population size is greater. In a 5-year intervention model the initial FRD population was shown to be changed by 1.53, 0.89, 0.56, 0.47 and 0.41 times for 10, 30, 50, 60 and 70% annual female dog spaying, respectively. The corresponding figures for FRD population change in 10-year period were predicted as 2.03, 0.81, 0.32, 0.22 and 0.16 (Table 2).

**Table 2:** Dog population model predictions according to five scenarios of female dog sterilization and three different levels of carrying capacities, 16,592 (K1), 24,200 (K2) and 55,000 (K3).

Annual spaying rate of female dogs (%)		No of dogs after 5 years (95% UI)	5-year population change compared to the baseline population	5-year population change compared to the projected population with no intervention	No of dogs after 10 years (95% UI)	10-year population change compared to the baseline population	10-year population change compared to the projected population with no intervention
0	K1	11,807 (7706, 14,631)	1.74	1.00	14,468 (8818, 16,270)	2.13	1.00
	K2	13,665 (7744, 18,515)	2.02	1.00	19,376 (8922, 24,026)	2.86	1.00
	K3	16,163 (7703, 24,555)	2.38	1.00	31,343 (8800, 50,129)	4.62	1.00
10	K1	9294 (6261, 11,589)	1.37	0.79	10,947 (5829, 13,679)	1.61	0.76
	K2	10,371 (6195, 1392)	1.53	0.76	13,766 (5693, 19,356)	2.03	0.71
	K3	11,718 (6066, 1738)	1.73	0.72	19,162 (5447, 33,596)	2.83	0.61
30	K1	5713 (4282, 6886)	0.84	0.48	4960 (2610, 6958)	0.73	0.34
	K2	6021 (4121, 7700)	0.89	0.44	5488 (2439, 8456)	0.81	0.28
	K3	6362 (3925, 8709)	0.94	0.39	6191 (2247, 10,786)	0.91	0.20
50	K1	3766 (3155, 4281)	0.56	0.32	2123 (1323, 2875)	0.31	0.15
	K2	3786 (2955, 4518)	0.56	0.28	2151 (1198, 3119)	0.32	0.11
	K3	3794 (2739, 4782)	0.56	0.23	2177 (1071, 3409)	0.32	0.07
60	K1	3233 (2823, 3581)	0.48	0.27	1504 (1025, 1958)	0.22	0.10
	K2	3175 (2609, 3671)	0.47	0.23	1470 (912, 2032)	0.22	0.08
	K3	3102 (2386, 3766)	0.46	0.19	1430 (805, 2109)	0.21	0.05
70	K1	2930 (2627, 3194)	0.43	0.25	1178 (855, 1483)	0.17	0.08
	K2	2807 (2391, 3173)	0.41	0.21	1111 (748, 1471)	0.16	0.06
	K3	2672 (2152, 3148)	0.39	0.17	1041 (647, 1455)	0.15	0.03

*UI* Uncertainty Interval.

## Discussion

The present study explored demography and dynamic modeling of free-roaming dog population in the city and suburbs of Kerman. FRD population size estimation as well as dynamic modeling of dog population under different female neutering interventions were also investigated. Free-roaming dog population management is a global challenge affecting many countries around the world. Uncontrolled FRD population can result in major public health problems and poor animal welfare^5^. To address this challenge, a comprehensive situation analysis is needed at local level to understand the nature and extent of the problem. Understanding the dynamics of dog population and investigating FRD population demographics are essential for a successful dog population management system.

Present study estimated the FRD population at 6781, with a density of 1.2 dogs per 100 people. The population size of FRDs varies widely in different communities around the world with an estimated variance of > 500 dogs per 100 people^37^. The literature search on major scientific databases showed the dog population per 100 people ranges from 0.2 to > 20 in different countries. Table 3 summarizes the dog population density in different parts of the world.

**Table 3:** Dog population density in different parts of the world presented as the number of dogs per hundred people.

Dogs per 100 people	Region (dogs per 100 people)	References
< 1	United Kingdom (0.18), Turkey (0.25)	^73,74^
1–5	Italy (1.4), , Iran (1.2) , China (2.07), India (3), Urban Africa (4.7)	^37,73,74,^, present study
5–10	Sri Lanka (5–10), Indonesia (6.25), Bhutan (6.7), South Africa (6.7–9), Rural Asia (6.9), Central/Eastern Tanzania (7.1)	^37,50,73^
10–15	Mexico (12.5), South Africa (13), Kenya (13), Urban Asia (13.3), Rural Africa (13.5), Canada (14.3), Thailand (14.9)	^5,21,37,73^
15–20	Zimbabwe (15.4), Tanzania (15.8), Peru (16.7), Sri Lanka (17.5), Argentina (18.3), Japan (19.2), Bali (19.2), Mauritius (20)	^5,21,37^
> 20	Nepal (21.2), USA (22.4), Timor-Leste (23), Bolivia (25), the Philippines (26.3), Bahamas (33–36), Mexico (43.4)	^21,37,38,75^

Dog density is an important index for any dog population management program. In the present study dog population density in the suburbs was significantly higher than the city. One explanation can be the greater carrying capacity in the suburbs than the city. In the suburbs of Kerman, lower human residential areas, vast areas of useless grounds and frequent garbage dumps are among the main factors providing larger carrying capacity for FRDs. In the city useless grounds and vacant lots are less available and dense residential areas provide relatively less carrying capacity in the city than the suburbs. Moreover tolerance of FRD population is higher in less developed suburb areas than the city. Interactions between the dog population of the city and suburbs is essential, however the nature and extent of population exchange is poorly documented^38^. Personal observations indicate that dogs regularly infiltrate from the suburbs into the city in search of food resources. The movement of dogs is known to depend on special characteristics of the geographical landscape and availability of food^39^.

We found a significant male to female ratio (3.2:1) among the FRD population. This male-skewed ratio is in accordance with the findings of other studies in different regions and countries^25,40–47^. Significant differential mortality between male and female dogs has been documented in several studies, because the female FRDs are more probable to develop malnutrition, disease and postpartum complications^25,47,48^. Studies of free-roaming dog populations in India, Indonesia, Chile, Nepal, South Africa and Spain reported male to female ratios ranges from 1.51 to 2.45^25,40–46^. Lower male to female ratios have been reported from Bhutan (1.31:1) and from Panchkula (1.34:1) and West Bengal (1.37:1) in India, possibly due to the ongoing animal birth control programs in the areas^25,49,50^. It has been shown that the neutered female dogs tend to live longer than the intact dogs^51^. Nevertheless other parameters including emigration, abandonment and ownership status might play a role in the sex structure of FRD populations in different regions, as it has been shown that there is a human preferences for adopting male dogs, and higher chance of female dogs to be abandoned^47,48^. Multivariate analysis showed females are 1.6 times more likely to be seen in the city than the suburbs. This may be due to the longer roaming distance of male dogs than females, for example, Durr et al. found that male dogs are more likely to roam out of the city^52^.

More than two third of dog population were adult, however the age structure of FRDs was not found different between the city and suburbs. Similar findings were reported in other countries such as India, Indonesia, Bhutan and Nigeria^25,42,50,53^. This is partly due to the higher mortality of pups and young dogs compared to the adult population^54^. Also it should be noted that pups do not usually show up in the streets and this may be partly due to the higher mortality, and puppies may be less likely to be observed by the research team.

The body condition of the dogs were found as ideal in more than 80% of FRDs. Interestingly the likelihood of sighting an ideal or overweight dog in the city was 14.9 times higher than in the suburbs. This is a strong indication of much higher food availability for dogs in the city compared to less developed areas of the suburbs. High proportions of free-roaming dogs with good body conditions are reported in Bhutan (92.7%) and India (68%). As stated in the studies, there are open garbage bins in the city, and dogs are frequently looking for food in the garbage^12,25^. In India open garbage dumps near residential houses was shown to be associated with stray dog population and the risk of rabies transmission^55^. However, further studies are needed as there is no hard evidence is available to show significant changes in free roaming dog population following solid waste management. It should be noted that controlling dog population through waste management is considered inhumane and solid waste management is only a minor part of a more comprehensive dog population management strategy, as free roaming dog populations supposed to be minimized through main measures such as sterilization and reduction in abandonment^56,57^. It should be noted that weight gain is also commonly seen as post spay-neuter in both males and females^5^, however no comprehensive FRD population management has ever been implemented in Iran and systematic TNVR (CNVR) programs have not been established in the country, although some sporadic efforts are being made by municipalities in large cities. In Kerman a dog shelter has been provided by the municipality and a small proportion of FRDs have been neutered monthly, nonetheless the neutered dogs will not be released in their original locations. Therefore the weight gain does not seem to be affected by this factor.

Analysis of the locations where dogs were observed within the city, showed that about half of the dog population were spotted in the vacant lots. The older parts of the city with ruins of abandoned old buildings were found as one of the hotspots of free roaming dog population within the city. In these locations the residents and shopkeepers, dump their garbage in the nearby vacant lots. These locations provide a safe shelter for FRDs for rest and reproduction. City officials including city council and the municipality should manage the abandoned buildings and vacant lots through appropriate municipal development plans including vacant lots closure and renovating the abandoned buildings. Simultaneously, as dogs are likely to be found in these areas, priority should be given to TVNR campaigns targeting these areas.

As shown in our spatial analysis high density of FRD population was observed in less developed areas around the circumference belt of the city where municipal services are weak and sufficient food and space is available for high number of dog population to live and breed. Therefore DPM plans could be targeted to these zones. Rapidly growing dog population is strongly associated with poor urbanization and household waste disposal^58^. It has been estimated that solid waste production by urban people is two- to threefold more than that of the rural residents^56,59^. Globally, 55% of people reside in an urban environment. The United Nations estimates this will increase to 68% by 2050, leading to a tremendous increase in solid waste production^60^. Dynamic modeling of FRD population could be improved by including parameters related to solid waste management in the urban areas.

Municipal development plans for the poor urban zones and the suburbs are advantageous for implementation of dog population management in the areas. Free-roaming dogs prefer these less populated areas for resting and breeding. As shown in our study a large proportion of FRDs were observed in vacant lots and abandoned buildings, therefore improvements in urban development including improved municipal services, construction of new residential/commercial blocks in the abandoned areas and improved transportation services lead to a reduced chance of breeding and relocation of the dogs to the more remote areas outside the city.

This study presented a system dynamics model of FRD population in Kerman. The model predicts that under existing conditions and no intervention, FRD population increases by 2.02 and 2.86 times in 5- and 10-year periods, respectively. There is a consensus that removing dogs is neither ethical nor effective in the management of free-roaming dog population and fertility-based measures is the key intervention for dog population management. Using agent-based models, Yoak et al. demonstrated that lethal methods carried out on FRD population in the city of Jaipur, India, skew dog population towards younger dogs, and increase human–dog conflicts^33^. The main reason to develop such models is to have a measure with which we compare the achievements of different female dog neutering interventions and allows us to understand the dynamics of FRD populations.

Figure 3 shows the simulation results of the annual spaying rate of female dogs run for a period of 10 years. Compared to the base run, dog population is reduced in all five scenarios, indicating that management options in all scenarios can be effective. Comparing different annual female neutering coverages, a higher female neutering coverage is more effective at reducing the dog population size, however as it was shown by Belsare and Vanak, managerial and financial limitations are major challenges in reaching high levels of neutering coverage^34^. In Iran no study and/or systematic program have been performed on sterilization-based dog population management and no data is available to compare with. Unfortunately in most DPM studies the number of the neutered free-roaming dogs is unknown and few studies have measured the impact and efficacy of field intervention programs in the urban settings. However, several modeling studies have been carried out on changes in dog population size. In Brazil dog population dynamics was modeled and the findings showed the need to intensify the current sterilization efforts. However the study was particularly focused on owned dog population and the impact of fertility-based interventions^29^. WSPA found that, following an ABC program in Jaipur, India, the dog density declined by one third between 1997 and 2002. In a rural setting in Bali, dog population in targeted villages was reduced by over 50% when 75% of the dogs were spayed or neutered^5^.

In the urban setting, a couple of studies have investigated fertility-based interventions on owned dog populations. In Mexico, the model developed by Kisiel et al. offered that, regardless of dog age and sex, following 8.6–34.5% annual sterilization, the dog population size after 20 years was reduced between 14 and 79% compared to the absence of intervention. They concluded that sterilization of only young, female dogs has the greatest impact on population reduction of 90–91%^32^. Another study on owned dog population in Italy estimated that to stop population growth, a sterilization rate of > 55% is needed and concluded that only 26% sterilization of female young dogs is adequate to halt dog population growth. Our findings estimated that sterilization rates less than 30% is not sufficiently effective to produce tangible reduction in FRD populations. It should be noted that the present study focused on unowned FRDs and it is difficult to compare the findings with owned dog populations. The projections of the model have shown that at least at a local context, 50% female dog sterilization in a 5 and 10 year period, significantly reduced free-roaming dog population by 0.44 and 0.68, respectively, compared to the baseline population (Table 2). However, ICAM recommends 70% annual female dog sterilization as an optimum coverage for an effective humane dog population management^16^. Seventy percent annual female dog sterilization has been achieved in Dehradun, India. This program reported a 35.6% decline in dog density over 2.5 years by comparing the total number of dogs seen on surveys along standard routes^61^. Sustainability is a key for any successful humane DPM program. As found by WSPA, while there were rapid increases in the proportion of female dogs sterilized (10–60%) in the first 3 years of the program, much slower scale-up to about 75% was recorded over the next 6 years^5^. Also to protect FRDs welfare and to prevent possible negative impacts of DPM programs, particular attention should be made on dog health and welfare issues. It is essential that successful dog population management programs be focused on reducing target population and consider physiological, psychological and physical needs of individual dogs^11,62,63^^.^

As with any model-based analysis, our study faced some limitations. We used some of the main parameters of the model from the literature as well as from studies carried out in other localities. This may limit the findings of the study. Extensive field studies in different parts of the world are required to provide further hard evidence of the demography, population dynamics and free-roaming dog population management in developing countries. The study did not compare other interventions (e.g. other dog population management methods, or targeting age groups, or male neutering) and this could limits the conclusions. In addition, based on our field observations and expert opinions, as dog ownership is not encouraged in the country, the present study assumed FRDs as a closed population in which immigration/emigration, rehoming/adoption, and abandonment have not taken place. Also sterilization interventions were assumed to be equally distributed across the city, therefore in those areas with higher levels of neutering coverage, it might be harder to find, catch and neuter dogs, and this could limit interpretation of the results. With using Lincoln–Petersen estimator, we could not quantify level of uncertainty in our size estimations. In the present study two transects, one capture and one recapture survey, were conducted to estimate dog population. In the case of multiple captured data, it was possible to use log-linear models and to take possible interactions into account. Moreover it gives us more confidence in population size estimation. As we used these population size estimations in our statistical analysis, further uncertainty is expecting to occur in some statistical associations. However, current ICAM guidelines recommend two-round surveys in developing countries^64^. Regarding the large study area, coordinating the complex operation involving several people, facilities, supplies and costs was a serious challenge for repeating the work in more than two rounds.

## Conclusion

Free-roaming dog population is a major challenge in many developing countries. Understanding the dynamics of FRD populations using modeling methods is critical for planning and implementation of dog population management programs. This study described the characteristics and dynamics of FRD population using modeling methods in order to determine the most appropriate spaying strategy under different levels of coverage. The projections of the model showed that at least at a local context, 50% female dog sterilization in a 5 to 10 year period, significantly reduced free-roaming dog population. Further investigations on dog population dynamics in other major urban areas of the country are required to improve our knowledge on the nature and extent of the challenges associated with uncontrolled dog population. Controlling dog population size is likely to result in reduced public health risks and improved animal welfare in developing countries.

## Methods

### Study location, design and data collection

In accordance with the guidelines of the World Society for the Protection of Animals (WSPA), Kerman city map with a scale of 1/100,000 was obtained. Kerman, as the capital city of Kerman province, is situated in the southeastern part of Iran, with an altitude of 1755 m. Kerman is situated at 30.29 latitudes and 57.06 longitudes. The city has a total population of 547,558 people and an area of 220 km^2^ (http://amar.sci.org.ir). The city was divided into 100 consecutive blocks, each block covers a surface area of approximately 2 km^2^. The blocks were assigned proportionately and the blocking was started from the city center and each block was identified with one of the four colors i.e. green, blue, red and yellow (Fig. 1). No adjacent blocks were assigned the same color. Finally, one color was randomly selected and 25 city blocks were identified for the study^65^. Also all 15 suburbs surrounding the city were included in this study, giving a total of 40 blocks.

For each block photography-based sight-resight surveys were performed by two trained teams of three persons, each team consisted of a car driver and two observers conducting photography and data collection. Briefly, by driving at a constant speed of ~ 20 km/h following a route (survey-track), predefined by using Google Maps, each block was scanned in early morning between 5:30 and 7:30 am when the free roaming dogs are active and visible after nighttime activity^4,12,30,66^. A checklist was designed for this study, consisted of general information including date, time, block code, starting point, end point, capture/recapture, temperature, humidity, wind speed, weather condition and the distance traveled. Every free-roaming dog that was observed was recorded. For each dog demographic parameters including age (pup, young, adult), sex (male, female, unknown), female status (pregnant, lactating, non-pregnant), body condition score (BCS), mobility/behavioral status (resting, running, feeding, mating, walking, and socializing) were visually determined by an expert in each team and the GPS coordinates and location were recorded using Google Maps in a mobile App. Scoring of FRDs body condition was performed on a 1–5 scale, according to the instructions described by ICAM^67^. The dogs were photographed using a professional camera (Nikon D5300 with a 18–140 lens and focal length of 140 mm). Two surveys were conducted in each block. The second survey was conducted one week after the first survey on the same time and track. After collecting field data, an ID number was assigned to each dog in a spreadsheet. Image analysis was performed in team meetings by comparing dog photos taken in each block according to different appearance characters and specific physical markings of the dogs to establish whether each dog was recaptured or only seen once during the initial capture.

Univariate and multivariate logistic regression models were used to calculate odds ratios of different variables relating to FRD population dynamics. The data analyses were performed by SPSS ver. 21. Multivariate logistic regression model was constructed based on the records from those dogs that all FRD population data were available for demographic variables including sex, age, body condition, and social organization (single, pair and pack). Where this information was missing, these individuals were excluded from the analysis. City and suburb dog population were considered as the outcome variables and sex, age, body condition and social organization as the predictor variables. At the first step, we assessed the univariate association between each variable and the outcome. To develop the multivariate models, we offered all variables to the multivariate logistic regression model. In a complementary analysis, the multivariate model was constructed in conjunction with Backward Elimination (B.E.) variable selection algorithm. This was performed to tackle potential multicollinearity problem.

Chi-square test for independence was used to analyze categorical variables including location, sex, age groups, body condition score and social organization.

### Estimation of free roaming dog population

To estimate the total population of FRDs in the city and suburbs, the number of dogs sighted in the first and second surveys as well as the number of dogs re-sighted in the second survey according to Lincoln–Petersen’s formula with Chapman’s correction^68^

1 $$N = \frac{{\left( {n1 + 1} \right)\left( {n2 + 1} \right)}}{{\left( {r + 1} \right)}} - 1$$

where n1 and n2 are the numbers of dogs sighted in the first survey and the second survey respectively, r is the number of dogs in the first survey that is re-sighted in the second survey, and N is the total population size. Extrapolation was made for the city population and the estimated number of dogs in the 25 city blocks was extrapolated to city-wide estimate. We defined free-roaming dogs as the dogs sighted in public areas, without any human companion and not currently controlled directly or indirectly by humans.

### GIS data analysis

The GPS coordinates reported for each dog during capture-recapture, were used to perform GIS analysis and to demonstrate density and spatial distributions of dog population. The distribution and density of FRDs were plotted on map using ArcGIS 10.2 software using the Kernel technique^39^.

### Model parameters and validation

Parameters affecting the dynamics of the dog population were identified in the literature. The main parameters identified were birth rate, death rate, immigrant/emigration, rehoming, weather, food resources and abandonment. Appropriate values for some of the parameters were found and extracted from the literature (Table 4). For other parameters, we conducted focus group discussions with two animal welfare NGOs, 15 municipal authorities, 14 veterinarians, and five kennel owners, to reach decisions about the appropriate values. The focus group discussions were conducted face-to-face or via phone calls. The team was introduced and the purposes of the study were explained before the discussion. Three persons in the research team (SS, SN and MFH) took notes. Subsequently the main points raised by the interviewees were extracted and discussed in a lab meetings.

**Table 4:** Definition of the parameters and the values used in dynamic modeling of free-roaming dog population in Kerman and suburbs.

Parameter	Definition	Value	Distribution	Unit	References
Puppy&Young	Number of puppy and young dogs (< 12 months) in the study area	1547	Fixed	No. of dogs	Present study (fieldwork)
Adult	Number adult dogs (12–60 month) in the study area	5234	Fixed	No. of dogs	Present study (fieldwork)
Q	The number of litters per year	N (1, 0.01)	Normal	No. of dogs	^70^
Np	The total number of puppies per litter	(1–3.6)	Uniform	No. of dogs	^49,70^
B	Finite birth rates	(F/M)*Q*Np	Fixed	No. of dogs/year	^70^
M	Finite mortality	N (0.23, 0.01)	Normal	No. of dogs/year	^70^
A	Instantaneous birth rates	(B/(B − M)) * ln(1 + (B-M)	Fixed	No. of dogs	^70^
Sterilization. Rate	Sterilization rates of female dogs	10, 30, 50, 60 and 70% annual sterilization of female dogs	Fixed	Percent	Present study (expert opinion)
Female.W	Number of female dogs that were spayed	Female * Sterilization. Rate	Fixed	No. of dogs	Present study (calculated)
Female.F	Number of female dogs that weren’t spayed	Female-Female.W	Fixed	No. of dogs	Present study (calculated)
Intact female	The ratio of female dogs who weren’t spayed	Female.W/Female	Fixed	No. of dogs	Present study (calculated)
Birth. Rate	Annual birth rate	A*(1 − Intact female)	Fixed	No. of dogs/year	^70^
“F/M”	The ratio of female to male dogs	0.3125	Fixed	No. of dogs	Present study (calculated)
Female	The number of female dogs	Adults*(F/M)	Fixed	No. of dogs	Present study (fieldwork)
Male	The number of male dogs	(1 − F/M)*Adults	Fixed	No. of dogs	Present study (fieldwork)
Birth	Number of dogs born	Birth rate * (Male + Female.F)	Fixed	No. of dogs/year	Present study (calculated)
D	Instantaneous mortality rates	(M/(B − M)) * ln(1 + B − M)	Fixed	No. of dogs/year	^70^
Death1	The number of puppy and young dogs that died over a year	("Puppy&Young"*D*kt) + ("Puppy&Young"*QS*"Birth.Rate")	Fixed	No. of dogs/year	Present study (calculated)
Du1	The average duration that dogs move from Puppy-Young to Adult	1	Fixed	Year	Present study (expert opinion)
D1	The number of dogs move from Puppy-Young to Adult	Puppy-Young*Du1	Fixed	No. of dogs/year	Present study (calculated)
Death2	The number of Adult dogs that died over a year	(Adult*D*kt) + (QS*"Birth.Rate"*Adult)	Fixed	No. of dogs/year	Present study (calculated)
QS	Number of female dogs that were spayed	("Female.W"/(Population*"F/M"))	Fixed	No. of dogs	Present study (calculated)
K1	The maximum population that can exist in a given particular environment	16,592	Fixed	No. of dogs	^34^
K2	The maximum population that can exist in a given particular environment	24,200	Fixed	No. of dogs	^71^
K3	The maximum population that can exist in a given particular environment	55,000	Fixed	No. of dogs	^70^
Kt	The maximum population that can exist in a given particular environment	1 − (Population/k1) 1 − (Population/k2) 1 − (Population/k3)	Fixed	No. of dogs	^34,70,71^
BI	Number of dogs born	"Birth.Rate"*(Male + "Female.F")*Kt	Fixed	No. of dogs	Present study (calculated)
DI	The number of Adult dogs that died over a year	Death1 + Death2	Fixed	No. of dogs/year	Present study (calculated)
Population	Number of dogs in the study area	BI-DI	Fixed	No. of dogs	Present study (calculated)

**Figure 4:**
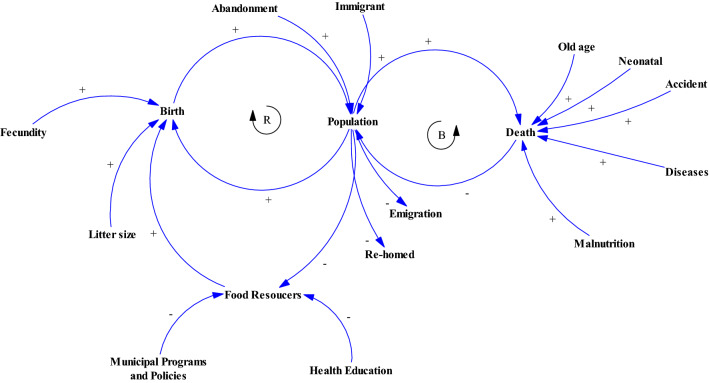
Causal Loop Diagram (CLD) showing parameters with major effects on free-roaming dog population. The arrows demonstrated cause-and-effect communication between a couple of variables, when two variables are linked in a CLD. When the two variables move in the same way this was represented by a “+”, and the reverse is indicated by a "−". Positive feedback loops showed by “R” while negative feedback loops showed by “B”.

After reviewing the literature, the conceptual dynamic model and Causal Loop Diagram (CLD) were constructed by Vensim™ ver. 6.4, (Ventana Systems Inc., UK) and optimized based on the regional settings (Fig. 4). Using Vensim™ with a stock and flow approach, a population dynamic model was developed as follows: the population variable was considered as stock, and the birth and death variables as flows, and birth, death rates and, carrying capacity were considered as auxiliary variables^14,69^. Weather, food resources and rehoming/abandonment were not used in the model and no immigration and emigration was assumed in the population. We used three different scenarios based on three levels of carrying capacity (low, medium and high) calculated based on the works published by different workers^34,70,71^. The lower level of carrying capacity (K1) was calculated based on a human:dog ratio of 33:1 in Belsare and Vanak. Having 547,558 human population in Kerman and suburbs, K1 was calculated as 16,592 dogs. The medium and high levels of carrying capacity (K2 and K3) were calculated based on the dog density of 110 dog/km^2^ in Kitala et al. and 250 dog/km^2^ in Amaku et al., respectively. Having 220 km^2^ surface area of Kerman city and suburbs, we estimated K2 and K3 at 24,200 and 55,000 dogs, respectively.

Using the following formula, the population can be calculated at any time:

2 $$\frac{d \, Population}{{dt}} = \left( {{\text{Birth.Rate}}}*\left( {{\text{Male}} }+ {\text{Female.F}} \right)*{\text{kt }} \right) - \left( {{\text{Death}}}1 + {\text{Death}}2 \right)$$

As the main aim of the study was to predict dynamics of FRD population in a 5- and 10-year period, we considered different scenarios including natural growth of dog population without any intervention as well as scenarios based on different spaying rates of FRDs. Accordingly six different scenarios were defined for predicting dynamics of FRD population after 5 and 10 years based on no intervention and 10, 30, 50, 60 and 70% annual sterilization of female dogs.

**Figure 5:**
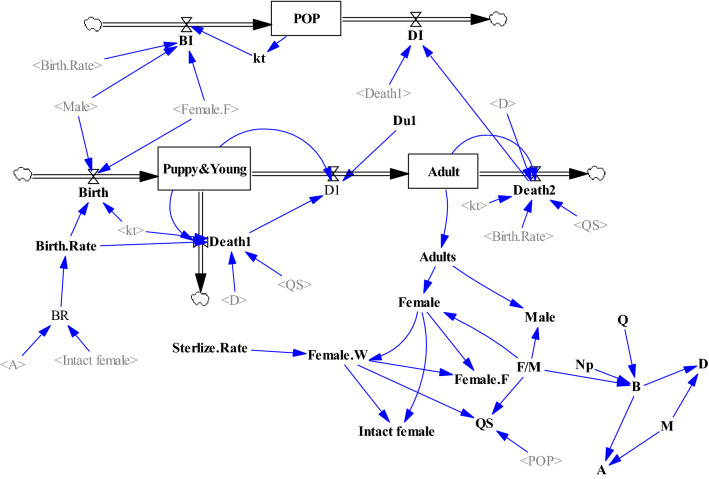
Model of the dynamics of dog population. Dynamic modeling of free-roaming dog population with implementation of animal birth control program.

As shown in Fig. 5, dynamic modeling was improved using the following data obtained in the field: No. of male and female dogs, age-specific dog population i.e. puppies/young dogs (0–12 months) and adult dogs (> 12 months). Annual population changes and differential equations defined for different age groups are shown in Eqs. (3) and (4).

3 $$\frac{{\text{d Puppy and Young}}}{{{\text{dt}}}} = \left( {{\text{Birth.Rate}}}*\left( {{\text{Male}}} + {\text{Female.F}} \right)*{\text{kt}} \right) - \left( \left( {\text{Puppy}} \& {\text{Young}}*{\text{D}}*{\text{kt}} \right) + \left( {{\text{Puppy}}}\, \&\, {\text{Young}}* {\text{QS}} *{\text{Birth.Rate}} \right) \right) - \left( \left( {\text{Puppy}}\, \&\, {\text{Young}}*{\text{Du}}1 \right) - \left( {{\text{Death}}}1 \right) \right)$$

4 $$\frac{{\text{d\, Adult}}}{{{\text{dt}}}} = \left( {\left( {{{{\rm Puppy}\,\&\, {\rm Young*Du}}}1} \right) - \left( {{\text{Death}}1} \right)} \right) - (\left({\rm Adult*D*kt} \right) + \left( {\rm QS* ``Birt".Rate *Adult} \right)$$

Input parameters used in the dynamic modeling were obtained from the literature and the field survey (Table 4).

Sensitivity analysis is performed in the model and the variables for which we had uncertainty, were statistically distributed and simulated by Monte Carlo method with 10,000 simulations. The upper and lower bounds were considered as 2.5 and 97.5, respectively and the mean was considered as the mean value of the uncertainty interval. Due to the lack of similar studies on dog population estimates in the country, the prediction model was manually validated by using the data either obtained from the field in the present study or the expert opinions^72^. We validated the model parameters according to the expert views. We had some focus group discussions with different experts in this topic and sought their ideas about different model components. The experts advised us on better choice of parameters' values compatible to the study area.

### Ethical approval

The project was approved by the ethical review committee of Kerman University of Medical Sciences (IR.KMU.REC.1399.091). All methods were carried out in accordance with KMU guidelines and regulations.
